# A Model for a Filling-in Process Triggered by Edges Predicts “Conflicting” Afterimage Effects

**DOI:** 10.3389/fnins.2018.00559

**Published:** 2018-08-17

**Authors:** Hadar Cohen-Duwek, Hedva Spitzer

**Affiliations:** Vision Research Laboratory, School of Electrical Engineering, Tel-Aviv University, Tel-Aviv, Israel

**Keywords:** afterimage effects, filling-in, diffusion, visual system mechanism, computational model

## Abstract

The goal of our research was to develop a compound computational model that predicts the “opposite” effects of the alternating aftereffects stimuli, such as the “color dove illusion” (Barkan and Spitzer, 2017), and the “filling in the afterimage after the image” (van Lier et al., 2009). The model is based on a filling-in mechanism, through a diffusion equation where the color and intensity of the perceived surface are obtained through a diffusion process of color from the stimulus edges. The model solves the diffusion equation with boundary conditions that takes the locations of the chromatic edges of the chromatic inducer (chromatic stimulus) and the achromatic remaining contours into account. These contours (edges) trigger the diffusion process. The same calculations are done for both types of afterimage effects, with the only difference related to the location of the remaining contour. While a gradient toward the inducing color produces a perception of the complementary color, an opposite gradient yields the perception of the same color as that of the chromatic inducer. Furthermore, we show that the same computational model can also predict new alternating aftereffects stimuli, such as the spiral stimulus, and the averaging of colors in alternating afterimage stimuli described by Anstis et al. (2012). The suggested model is able to predict most of the additional properties related to the “conflicting” phenomena that have been recently described in the literature, and thus supports the idea that a shared visual mechanism is responsible for both the positive and the negative effects.

## Introduction

This study concerns two non-classical afterimage illusions, both involving a chromatic stimulus i.e., a chromatic inducer that is presented for a short duration of time, and is then followed by the presentation of an achromatic remaining contour that may overlap with the inner or outer border of the chromatic region of the inducer. The location of this remaining contour, can determine whether the perceived filling-in color will be the same as, or complementary to, the chromatic inducer. Two famous examples of these phenomena are: the “Filling-in the Afterimage after the image effect” (van Lier et al., 2009), and the color dove illusion (Barkan and Spitzer, 2009, 2017; Macknik and Martinez-Conde, 2010). Both phenomena involve a filling-in process of surfaces between edges, and the effects are obtained with a narrow spatial inducing area and relatively short induction time. Since these two phenomena yield complementary perceived colors, derived from the very same inducer, we refer to them as “conflicting” effects.

**Figure 1:**
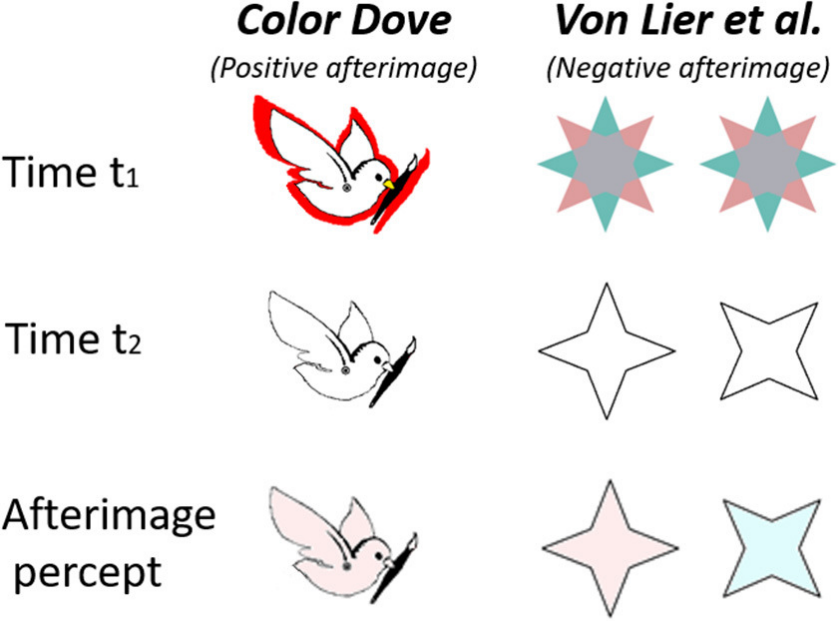
Demonstration of two “conflicting” alternating aftereffects, “The Color Dove illusion” (Positive effect, left column) and the Von Lier et al. Illusion (Negative effect, right columns). The first row shows the inducing stimulus, the second row shows the drawn contours presented at time t2, and the third row represents the resulting perceived afterimage.

In the “Filling in the Afterimage after the image” (van Lier et al., 2009) illusion, the inducing stimulus is a chromatic shape that may have two or more colors. After the chromatic inducing stimulus is removed, an outline contour matching one of the shape colors is presented. The complementary afterimage color perceived depends on the shape and the location of the drawn outline contour (van Lier et al., 2009), (Figure 1, second column). Since the color inside the contour in the perceived afterimage is complementary to the color of the inducing stimulus, we henceforth, refer to this illusion as a “negative effect.”

It should be noted that this negative effect is not a simple variation of the “classical” negative afterimage, where, when a stimulus is removed after a relatively long (20-30 seconds) exposure, the observer perceives the opposite chromaticity (complementary color DeValois and Webster, 2011). It should also be noted that the colors in the classical afterimage are perceived only in the retinotopic area that was induced.

In the color dove illusion (Barkan and Spitzer, 2009, 2017), the inducing stimulus is a shape surrounded by a colored area or strip (red in Figure 1, first row). After the chromatic inducing stimulus is removed, an outline contour matching the original inducing stimulus is presented (Figure 1, second row). This gives rise to the perception of an afterimage (Figure 1, third row) filled with a color similar to that in the inducing stimulus (although weaker), and not the complementary color as in the negative effect. Such an effect has also been reported with objects of different shapes (Hazenberg and van Lier, 2013). Since the perceived color inside the shape is similar to that presented in the inducing stimulus, we henceforth refer to this illusion as a “positive effect,” (Figure 1, first column).

A similar positive aftereffect was previously investigated by Anstis et al. (1978) who suggested that the positive chromatic afterimage effect is a result of the synergy of two known visual mechanisms: simultaneous contrast (Gerrits and Vendrik, 1970; Anstis et al., 1978) and colored afterimage (Daw, 1962; Wyszecki, 1986; Shimojo et al., 2001).

The alternating effects differ from a classical afterimage in their temporal and spatial properties. A classical afterimage requires a relatively long exposure time and a large spatial area of induction, in order to obtain a filling-in effect in a small region with the complementary color (Anstis et al., 1978). In the phenomena described here, preliminary results indicate that the positive effect is not abolished even if the area of the chromatic inducer is spatially thin (Hazenberg and van Lier, 2013; Barkan and Spitzer, 2017. This is in contrast to the explanation given by Anstis et al. (1978), since psychophysically, when the area of a chromatic inducer is thin, the effect of simultaneous contrast is not manifested (preliminary results). The positive and the negative effects are also distinguished from the classical aftereffect (Anstis et al., 1978), in their temporal properties. The duration of the alternating stimuli can be very short (500 ms), a period of time that is insufficient to obtain the classical afterimage effect (Anstis et al., 1978; van Lier et al., 2009; Barkan and Spitzer, 2017).

A further distinguishing characteristic of these phenomena is that, in addition to the temporal and spatial differences from the classic afterimage effect, the color in both the positive and negative effects is perceived in new areas that have not been induced or adapted previously (van Lier et al., 2009). It has to be noted that even though the positive and the negative effects share several common properties, they are still phenotypically different and therefore they can be seen as “conflicting effects.”

Hazenberg and van Lier (2013) investigated “alternating watercolors,” which have the spatial and the chromatic structure as of the classical watercolor stimuli. These types of stimuli can be considered as the positive and the negative stimuli, while the same classical watercolor stimulus is used as the chromatic inducer stimuli for both positive and negative aftereffects. In this case, the remaining contours are located at the inner or the outer contours of the chromatic edges of the inducer stimulus. The reported results (Hazenberg and van Lier (2013) indicated that the positive and negative effects were affected differently by a number of parameters including the luminance of the area inside the shape and the luminance of the remaining contour.

At present, the visual mechanisms responsible for the recently described positive and negative effects are still unknown and there are no successful computational models for the phenomena. This is less surprising in view of the fact that there remains a lack of consensus concerning the mechanism of even the classical afterimage, despite the wealth of research in the literature. The physiological mechanisms commonly proposed as responsible for the classical negative afterimages range from bleaching of cone photo-pigments to cortical adaptation (Williams and Macleod, 1979; Shimojo et al., 2001; Clair et al., 2007; van Lier et al., 2009; Zaidi et al., 2012; Webster, 2015; Zeki et al., 2017). A recent paper suggested a different mechanism to the van Lier et al. (2009) effect and attributed the filling-in process to the perception of transparency cue and cortical mechanisms (On and van Boxtel, 2017).

Additional recent research (Zaidi et al., 2012) has suggested that the classical and the negative afterimage effects are derived from the retinal ganglion mechanism, which yields the neuronal rebound effect. According to this mechanism, the ganglion neurons can fire bursts if inhibited and then released from inhibition (Spitzer et al., 1993; Grunfeld and Spitzer, 1995; Francis, 2010; Zaidi et al., 2012). It should be noted that while the rebound effect may modulate the creation of complementary colors, it cannot be responsible for the either the negative or positive effects in their entirety.

Previous computational models have been reported to describe both the complementary perceived color and the filling-in components (Grossberg and Todorovic, 1988; Francis and Rothmayer, 2003; Francis and Ericson, 2004; Francis and Schoonveld, 2005; Wede and Francis, 2006, 2007; Van Horn and Francis, 2008). These models were based on the original “Form And Color And Depth” FACADE) model (Grossberg and Mingolla, 1985), which described two main visual processing systems: a boundary contour system (BCS) that processes boundary or edge information, and a feature contour system (FCS) that uses information from the BCS to control the spreading (filling-in) of surface properties, such as color and brightness. According to the FACADE model, the filling-in stage requires the FCS networks to diffuse signals containing feature information about color and brightness across the surface, while boundaries in the BCS block the spreading.

The FACADE model and its variations succeed in predicting the afterimage effects of the MacKay modal complementary afterimages (MCAI) phenomena (MacKay, 1957; Vidyasagar et al., 1999). This effect involves sequential viewing of two orthogonally related patterns (the first one a constant pattern and the second one a flickering contrast reversal pattern). The result is an afterimage percept that is related to the first pattern (Francis and Rothmayer, 2003; Francis and Ericson, 2004; Francis and Schoonveld, 2005; Wede and Francis, 2006, 2007; Van Horn and Francis, 2008). A number of studies have examined the different spatial and temporal properties of the MCAI effect, for example the spatial and temporal frequency of the two gratings from the first and second presentations (Francis and Rothmayer, 2003), the gap width (Francis and Ericson, 2004), the split gratings (Francis and Schoonveld, 2005), duration between the two grating presentations and the blank presentation (Wede and Francis, 2006), attentional properties (Wede and Francis, 2007), and the role of the difference orientations of the constant and the flickering grating (Van Horn and Francis, 2008). Francis and colleagues confronted their computational model's prediction with the perceived results.

It should be noted that the MCAI and its variations discussed in these Francis papers are not necessarily related to the positive and negative aftereffects phenomena described in our current report. The main differences between the MCAI (MacKay, 1957; Vidyasagar et al., 1999) phenomena and the positive and the negative effects concern the different types of the stimulus components, at these two groups of effects. The stimulus differences related to the orientation gratings and contrast reversal flickering patterns used to produce the MCAI effect versus the chromatic shape of inducer and remaining contour that trigger the positive and negative effects. These differences in the type of stimuli might imply distinct mechanisms that involve additional different components, even though both models can basically be attributed to diffusion processes.

Francis (2010) applied a similar diffusion model to that described previously in Francis and Rothmayer (2003) in order to address the negative effect of van Lier's illusion (van Lier et al., 2009), and succeeded with the model's predictions. At a later stage, Kim and Francis (2011) conducted a series of psychophysical experiments designed to prove that a simple diffusion model (Francis, 2010) cannot account for the additional properties characterize the negative after effect. They tested the hypothesis, for example, that a contour traps the perceived afterimage color, by adding additional remaining contours. Their model simulations predicted that these additional remaining contours would block the spread of a color to the middle of the surface, Figure 4.

However, contrary to Francis's predictions (Francis, 2010), the results of the psychophysical experiments showed that additional remaining contours blocked color spreading only when they overlapped with the inducer edges, but not when they were drawn away from the inducer edges (Kim and Francis, 2011), Figure 4. More important to our discussion is the fact that FACADE model did not and cannot model the positive effect. In this study, we present a computational model that can predict both the negative and the positive effects, and postulate that these effects are derived from the same mechanism. We also test whether the model can predict additional afterimage phenomena beyond the two described effects.

## Model

**Figure 2:**
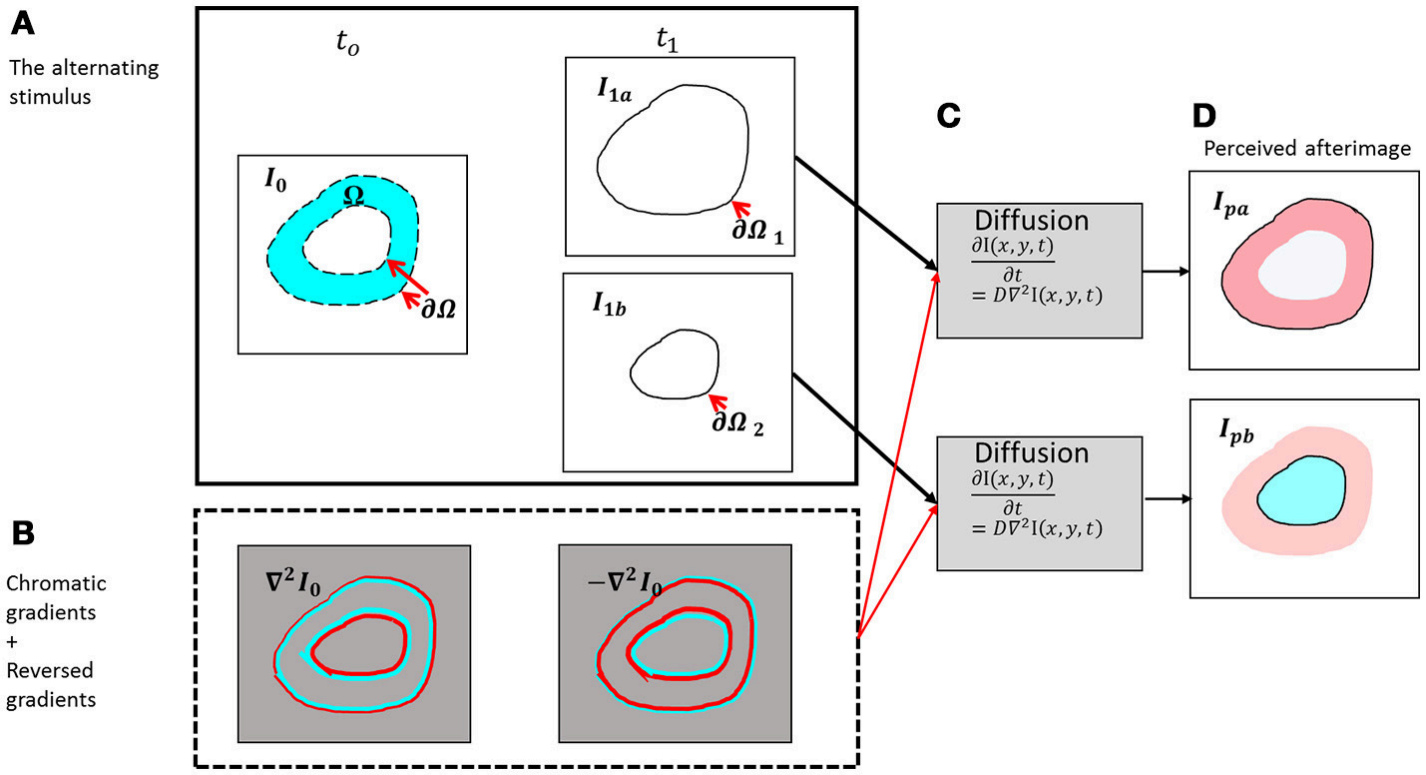
Schematic diagram of the presented model. **(A)** The chromatic stimulus (***I***_**0**_) at ***t***_**0**_ and the two options for the remaining contour [the outer contour - ***I***_**1a**_ (upper box) and the inner contour – ***I***_**1b**_ (lower box)]. **(B)** The chromatic gradients of the chromatic stimulus and their reversed chromatic gradients. **(C)** The diffusion process for the outer contour (upper box) and the diffusion process for the inner contour (lower box). **(D)** The perceived afterimages according to the outer remaining contour (negative effect, ***I***_***pa***_) and to the inner remaining contour (positive effect, ***I***_***pb***_).

The following sections describe a unified computational model that can predict the two known “conflicting” (opposite) phenomena, the positive “color dove illusion” and the negative “filling-in afterimage after the image” illusion. The model is also able to predict additional variations of the positive and the negative effects. We suggest here, that despite differences in their spatial and temporal properties, these two types of phenomena are produced by a very similar (mutual) mechanism. The model considers several crucial factors for the perceived temporal effects and these are presented in Figure 2.

### Model assumptions

The model is based on the following assumptions: (a) An edge triggers a diffusion process in its complementary color. (b) A contour can be a perceived contour and not necessarily a physical spectral gradient. (c) The diffusion process depends on the correspondence between the chromatic stimulus gradients and the remaining contours. (d). The positive and the negative effects are always present, while the dominant perceived color is determined by the location of the remaining contours.

### The stimulus: the chromatic inducer and the remaining contours

The input of the model is composed of two temporal components, the first one is a chromatic stimulus, *I*_0_ in Figure 2, and the second one relates to the remaining contours *I*_1*a*_ and *I*_1*b*_ in Figure 2. The remaining contours can appear in different possible locations, and these locations determine whether the perceived result will be a positive effect or a negative effect.

### Chromatic gradients

The building blocks of the model are designed to simulate components of the visual system, and in this case, the opponent and double-opponent receptive fields. The color coding opponent receptive fields encode color contrast, but not spatial contrast. In other words, the color opponent receptive fields are able to differentiate between colors, but cannot detect spatial gradients or edges (Barkan et al., 2008). The double opponent receptive fields, however, are sensitive to both spatial and chromatic gradients and have color opponent receptive fields both at the center and in the surround receptive field regions (Shapley and Hawken, 2011). This opponency in both spatial and chromatic properties produces a spatio-chromatic edge detector.

For the sake of simplicity, we compute the opponent response of the opponent receptive fields as color-opponent only, where, in this simplified case, each chromatic encoder contains the same spatial resolution. This is computed by an opponent color-transformation (Sande et al., 2010), Equation (1). This transformation converts each pixel of the image *I*_0_, in each chromatic channel R,G, and B into opponent color-space, via the transformation matrix *O* (Sande et al., 2010). *I*_*OPPONENT*_ = *OPPONENT*{*RGB*} as follows:

(1) $$\begin{array}{l} I_{OPPONENT}=\left(\begin{array}{c} O_{RG}\\ O_{YB}\\ O_{BW} \end{array}\right)=\left(\begin{array}{c} \frac{1}{\sqrt{2}}\frac{-1}{\sqrt{2}}0\\ \frac{1}{\sqrt{6}}\frac{1}{\sqrt{6}}\frac{-2}{\sqrt{6}}\\ \frac{1}{\sqrt{3}}\frac{1}{\sqrt{3}}\frac{1}{\sqrt{3}} \end{array}\right)\left(\begin{array}{c} R\\ G\\ B \end{array}\right) \end{array}$$

where *O*_*RG*_, *O*_*YB*_, *O*_*BW*_ are the new channels of the transformed image *I*_*OPPONENT*_. R, G, and B are the red, green and blue channels of *I*, respectively.

In order to implement the double-opponent response, *DO*, on an image, we subtract the surround, *O*_*surround*_, region of the receptive fields from its center, *O*_*center*_, at the same spatial location:

$$\begin{array}{l} DO=\;O_{center}-O_{surround} \end{array}$$

The structure of the double opponent receptive field can be seen as a filter which performs as a second derivative in both spatial and chromatic domains (Conway, 2001; Conway and Livingstone, 2006). For the sake of simplicity and clarity of the calculations, we use a discrete Laplace operator, *L*, which is commonly used as an approximation to the Difference of Gaussian (DOG) function (Marr, 1982). The discrete Laplace operator, L is (Weickert, 1998):

(2) $$\begin{array}{l} \text{L}=\left(\begin{array}{c} 0\;\frac{-1}{4}0\\ \frac{-1}{4}1\;\frac{-1}{4}\\ 0\frac{-1}{4}0 \end{array}\right) \end{array}$$

The responses of the relevant receptive fields, *DO*_*response*_, of the color coding receptive fields to the aftereffect stimuli are presented in Equation (3). The double-opponent *DO*_*response*_ response is calculated as a convolution of each opponent channel of *I*_*OPPONENT*_ with the discrete Laplace operator Equation (2).

(3) $$\begin{array}{l} {DO}_{response}\left(stimulus\;on\right)=\;\nabla^{2}I_{OP}\approx I_{OP}*L \end{array}$$

Figure 2B demonstrates the responses of the receptive fields to the original stimulus (Figure 2A) at time *t*_0_, Equation (3).

### The perceived gradients—the responses of the receptive fields to the aftereffects

The model suggests that after the chromatic stimulus disappears, the chromatic gradients obtain the opposite sign. We refer to this condition as “off response,” a term commonly used in electrophysiology (Kandel et al., 2012). The physiological mechanism behind this behavior is still a matter of discussion (Williams and Macleod, 1979; Spitzer et al., 1993; Shimojo et al., 2001; Clair et al., 2007; van Lier et al., 2009; Francis, 2010; Zaidi et al., 2012; Webster, 2015; Zeki et al., 2017). This response has also been termed the rebound response and a variety of models and mechanisms have been suggested to explain how this rebound phenomenon yields a reversed type of response (Spitzer et al., 1993; Grunfeld and Spitzer, 1995; Francis, 2010; Zaidi et al., 2012). Figure 2B demonstrates the responses of the simulated receptive fields before and after the chromatic stimulus is removed at times *t*_0_ and *t*_1_, Equation (4).

(4) $$\begin{array}{l} {DO}_{response}\left(stimulus\;off\right)=\;I_{OP}*\left(-L\right) \end{array}$$

In other words, in this case, the sign of the chromatic gradient, *DO*_*response*_, is reversed. Note that the disappearance of the chromatic stimulus, which causes the sign of the edge to be reversed, is in accordance to the model's assumption (section Model Assumptions, A). There are also experimental results that support this assumption (Zaidi et al., 2012).

This operation of edge reversal is realized in the model through reversing the sign of the DO receptive field responses, Equation (3). This reversed chromatic gradient triggers the diffusion process, Figure 2C, Equation (5).

### Filling-in as a diffusion process

The diffusion process is expressed by the diffusion (or heat) Equation (5), (Weickert, 1998). The model assumes that the suggested diffusion of the filling in process is similar to the physical diffusion where the signals spread in all directions, until “blocked” by contours or edges. This type of filling-in process is referred in the literature as the “isomorphic filling-in theory” (von der Heydt et al., 2003). The choice of such a type of filling-in infers that the borders (chromatic or achromatic) do not function primarily as blockers, but instead that the borders play a role as heat sources for the diffusion. When the direction of the diffusion spread is in the opposite direction (colliding) to that of an additional heat source, the spread will actually be blocked by the heat source. These principles are applied in our model through the famous diffusion equation (Weickert, 1998), as in the following equation:

(5) $$\begin{array}{l} \frac{\partial I\left(x,y,t\right)}{\partial t}-D\nabla^{2}I\left(x,y,t\right)=\;h_{c} \end{array}$$

where *I*(*x, y, t*) denote the image in a space-time location (*x, y, t*), D is the diffusion (or heat) coefficient, and *h*_*c*_ represents a heat source. The time course of the perceived image is assumed to be very fast, in accordance with previous reports (van Lier et al., 2009; Barkan and Spitzer, 2017). This time course is also termed “immediate filling-in” (von der Heydt et al., 2003).

Following this assumption, for the sake of simplicity, we can ignore the fast dynamic stages of the diffusion equation, and therefore compute only the steady-state stage of the diffusion process. Consequently, the diffusion (heat) Equation (5) is reduced to the Poisson Equation (6).

(6) $$\begin{array}{l} \nabla^{2}I\left(x,y,t\right)=-h_{c} \end{array}$$

## The chromatic edges and the remaining contours

In order to maintain and enhance and/or byproduct to trap this diffusion effect there is a “requirement” for a border. The model suggests that the chromatic diffusion can be “trapped” only when the achromatic remaining contour, ∂Ω_1_ Figure 2A, overlaps the original edges of the chromatic stimulus, *DO*_*response*_. Support for this assumption is also provided from the psychophysical results of Kim and Francis (2011).

Whether the reminding contour ∂Ω_1_, is an inner or an outer contour, for example (Figure 2), determines the perceived color of the effect. When the remaining contour is the outer contour, the reversed contour, i.e.; the complementary contour, [Figure 2A, Equation (4)] triggers a diffusion color that is complementary to the color of the inducer, i.e. red in the specific case of Figure 2B. The outer contour, ∂Ω_1_, determines that the fill-in color will be complementary to the inducer (negative effect), whereas the inner contour, ∂Ω_2_, determines that the fill-in color will be the same color as that of the inducer (positive effect). It has to be noted that the mechanism detects the chromatic edges, and does not treat the inner or outer edges separately. The configuration and the locations of the remaining contours, and not the model, determine the predicted perceived colors.

It is clear that a remaining contour that overlaps the chromatic gradient plays a role as a diffusion trigger and at the same time as a “blocker.” However, our preliminary results suggest that the original chromatic gradient, *DO*_*response*_, also plays a role as a diffusion trigger and “blocker,” even though it has a weaker effect when it does not overlap the remaining contours. This observation is also supported by findings of Hazenberg and van Lier (2013). They concluded that the chromatic border in the negative effect “apparently prevented the colored afterimage of the chromatic contour from spreading.”

This minor effect of additional blockage, derived from the chromatic edges, has been integrated into the model by applying different weight functions to each chromatic and achromatic border. The model assumes that the remaining contour also plays a role as an enhancer to the reversed chromatic edges, −*DO*_*response*_. Therefore, if the remaining edge, ∂Ω_1_, overlaps the original gradient edge (the chromatic gradients of the inducing stimulus, −*DO*_*response*_), it will enhance these chromatic edges. The mathematical expression of this role is expressed by the weight functions α and β:

(7) $$\begin{array}{l} \nabla^{2}O_{p}={-DO}_{response}\cdot \left(\alpha \partial \Omega_{1}+\beta \right),\;where\;\alpha >\beta \end{array}$$

where *O*_*p*_ is the perceived image in the opponent color-space (Sande et al., 2010) and α and β are constants, but can be further extended to be functions.

Solving Equation (7) yields a response to the perceived afterimage *O*_*p*_ given the reversed gradients −*DO*_*response*_(*x, y, i*), Equation (4), according to specific initial constraint. Figure 2D represents the perceived afterimage, *O*_*p*_, but with an additional technical stage of transforming the opponent color space *O*_*p*_ to the RGB color space, *I*_*P*(*rgb*)_, Equation (11).

The interpretation of the solution as suggested above is that a very similar mechanism is responsible for both the negative and the positive effects, although it is possible that the two phenomena do not stem from the exact same visual mechanism. The model may separate the positive and the negative effects to two channels. One channel is for the chromatic area, where the negative effect is more dominant, while the other channel serves the achromatic area, where the positive effect is more dominant. Since the negative effect is given by a response from the chromatic induced region, whereas with positive effect there is a perceived response to an area that has not been induced with color, we assumed that the weight function of the negative effect should be higher than the positive effect (Equation 10). This separation can be justified by analogy to the visual system. The existence of separated Magno, Parvo, and Konio visual pathways in the visual system suggests that separating chromatic and achromatic calculations in this way may be a true reflection of the visual system processing (Shevell, 2003).

We implanted the two separated channels for the positive and negative effects by calculating the diffusion Equation (5), separately for the chromatic and achromatic zones in the original image (*I*_0_). The positive effect *O*_*p, positive*_ occurs in the achromatic zones of the initial image *I*_0_, Figure 2A and the negative effect *O*_*p, negative*_ occurs in the chromatic zones of the initial image *I*_0_, Figure 2A. Accordingly, the equation is solved separately for the negative effect *O*_*p, negative*_ and for the positive effect, *O*_*p, positive*_, (see the section above). The simulation result is calculated as:

(8) $$\begin{array}{l} \nabla^{2}O_{p,negative}\left(\text{x},\text{y},\text{i}\right)=-{\text{DO}}_{\text{response}}\left(\text{x},\text{y},\text{i}\right)\cdot \left(\alpha \partial \Omega_{1}+\beta \right)\text{ in }\Omega \end{array}$$

(9) $$\begin{array}{l} \nabla^{2}O_{p,negative}\left(\text{x},\text{y},\text{i}\right)=-{\text{DO}}_{\text{response}}\left(\text{x},\text{y},\text{i}\right)\cdot \left(\alpha \partial \Omega_{1}+\beta \right)\text{in}\bar{\Omega } \end{array}$$

i = RG,YB,WB, where each opponent channel is solved separately.

(10) $$\begin{array}{l} O_{p}=\frac{O_{p,positive}+O_{p,negative}}{{max}_{all\text{\_}channels}\left\{I_{p,positive}\right\}+{max}_{all\text{\_}channels}\left\{I_{p,negative}\right\}} \end{array}$$

(11) $$\begin{array}{l} I_{P\left(rgb\right)}=OPPONENT^{-1}\;\left\{O_{p}\right\} \end{array}$$

where max_*all*_*channels*_{*I*} is the maximum value of all channels in the image *I* (max{*I*} *is a scalar*). α and β present the weights of the remaining contours and the chromatic stimulus edges, accordingly.

In order to calculate the perceived afterimage from both the negative *O*_*p, negative*_ and the positive *O*_*p, positive*_ effects, Equations (8–10), we need to define (a) boundary conditions, and (b) the initial values. We shall henceforth denote the inducing stimulus (the original color image) by *I*_0_, where Ω is an area in the image *I*_0_, and ∂Ω is the border of Ω, Figure 2A. *I*_1_ is the remaining contour image and ∂Ω_1_*or ∂Ω*_2_ are the remaining contours (the remaining boundaries, although the boundaries in *I*_1_ might be different from those in *I*_0_. Therefore, the chromatic edges, ∂Ω, do not necessarily overlap the remaining contours ∂Ω_1_*or ∂Ω*_2_). The boundary conditions of the perceived image *I*_*p*_ and the initial state (initial conditions) are chosen to be an achromatic white color on the output image border. Thus, the boundary condition is *O*_*p*_|_*border*_ = 1, Figure 2A and the initial image is a blank white image (R = G = B = 1). These conditions are selected in order to enable the generation of the perceived afterimage on a white image as in the original stimuli (Barkan and Spitzer, 2009; van Lier et al., 2009), Figure 2D.

## Results

### Simulation details

The simulations are produced by assigning the conditions (boundary conditions and initial values) as described above, and applying the Gauss-Seidel method. The simulations are solved in a similar way to that reported in “Methods for Solving Equations” (Simchony et al., 1990) or “Poisson Image Editing” (Pérez et al., 2003). The simulations are implemented by MATLAB software.

The only parameters in the model are α and β, which present the weights of the remaining contours and the chromatic stimulus edges, accordingly. The Parameters were chosen as following: α = 1.3 and β = 0.1, as results of trial and error. These parameters are constant for all the simulations; beside in the Supplementary Figure 1 where we intended to slightly enhance the result for demonstration.

### Model's simulation and predictions

The simulation results are divided into three parts. The first part presents the model predictions for both the negative and the positive afterimage phenomena, (Barkan and Spitzer, 2009; van Lier et al., 2009). The second part presents the predictions of the model for two remaining edge variations, as presented in previous studies (Francis, 2010; Kim and Francis, 2011). The third part presents the model predictions for additional aspects of the afterimage phenomenon, where one relates to the color perceived when the remaining edge of the image is not complete (open boundaries, spiral image), and the second relates to spatial averaging of colors, (Anstis et al., 2012).

### Negative and positive afterimages

**Figure 3:**
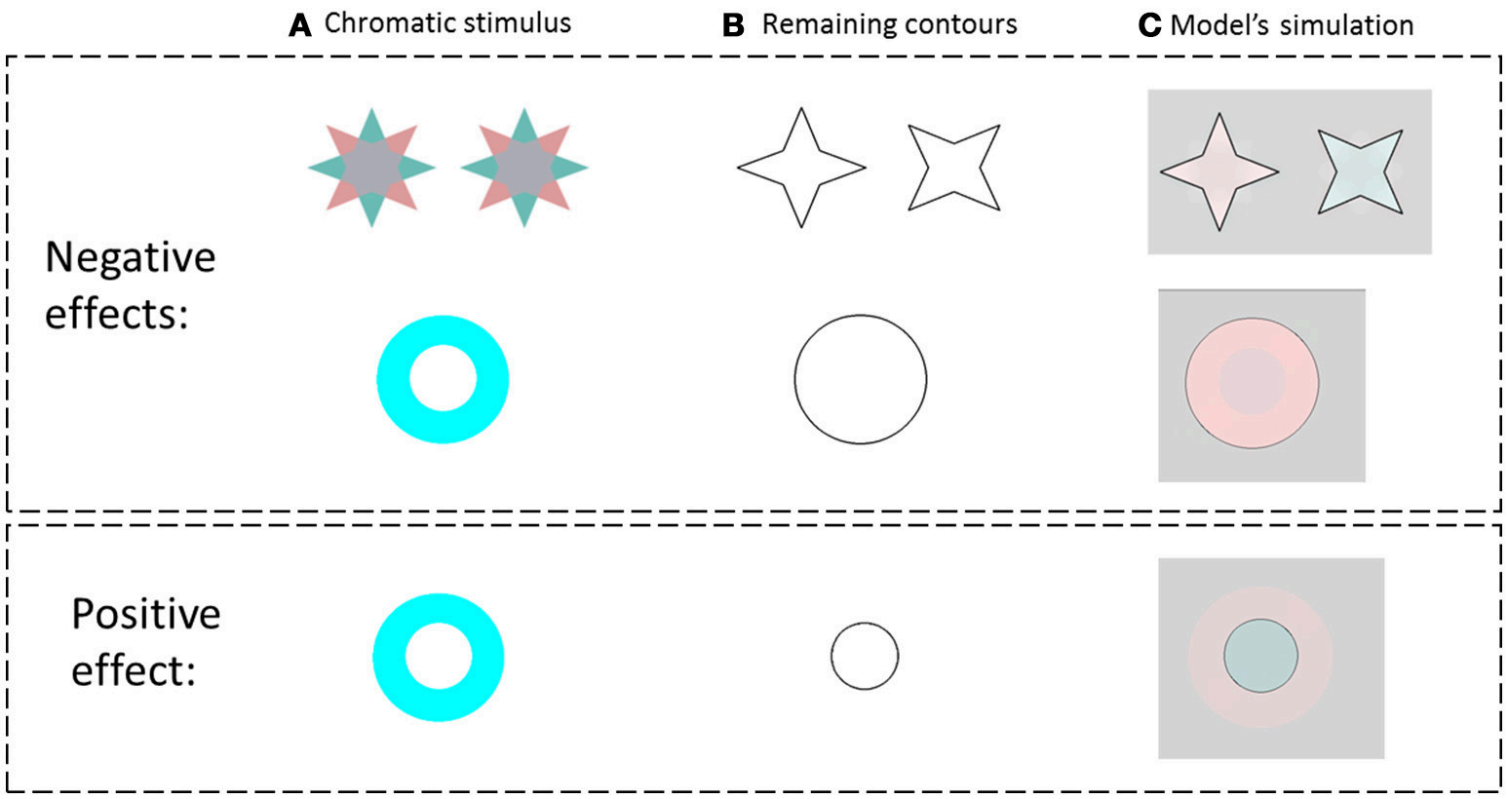
The model predictions for the negative and the positive effects. **(A)** The chromatic stimulus. **(B)** The remaining contours. **(C)** The simulation results. The first row presents the van Lier et al. (2009) stimuli, e.g., the negative effect (stars) and the model prediction for these stimuli. The second row, in the negative effect block, presents a general case of the negative effect, which displays only one chromatic inducer. The third row represents the model predictions for a general case of the positive effect (instead of the “color dove illusion”).

We tested the model on the same stimuli as in the study of van Lier et al. (2009) (Figure 3 first row), and for the general case of the chromatic stimuli *I*_0_, Figure 2. Figure 3 presents the model's predictions for a single colored ring as inducer (second and third rows). It can be seen that the model correctly predicts that the remaining contours can generate a negative or a positive effect depending on their location. Of note, the model correctly predicted the filling-in process of the achromatic area with respect to both negative and positive effects, with the results in accordance with the psychophysical findings reported previously (van Lier et al., 2009; Hazenberg and van Lier, 2013). Having different weight functions for the positive and negative effects (Equation 11) enables us to control the predicted effect of a stronger diffusion in the inner than in the outer region of the remaining contours (Figure 3). These studies showed that the perceived afterimage has the complementary color when the outer contour is remained, (Figure 3, second row), while the same color is perceived when the inner contour is remained (Figure 3, third row).

### The role of the remaining edges

#### Comparison to previous results

**Figure 4:**
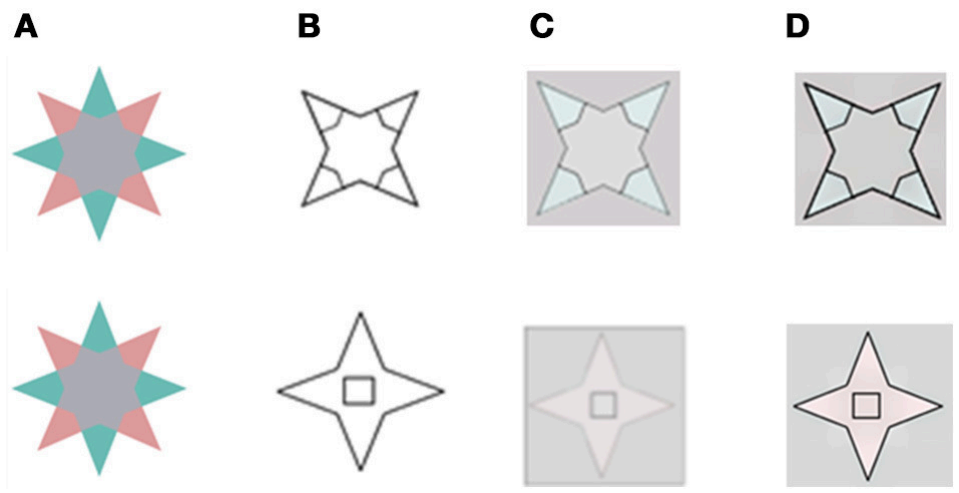
A comparison of our model's predictions to that of Francis for the two locations for the remaining edge that Francis tested experimentally. **(A)** The chromatic stimulus. **(B)** The remaining contours. **(C)** The simulation results as reported in (Francis, 2010; Kim and Francis, 2011). **(D)** The simulation results of the suggested model. In the first row, the inner drawn contours **(B)** overlap the chromatic gradients that exist in the inducing stimulus **(A)**. In the second row, the inner drawn contours do not overlap the chromatic gradients of the inducing stimulus. The results in **(D)** are in agreement with psycho-physical experiments (Kim and Francis, 2011).

We also tested our model on the same variations of the van Lier et al. (2009), stars stimulus that were tested by Francis (2010) and Kim and Francis (2011). These variations are related to the location and shape of the remaining contour. Figure 4 presents a comparison between the predictions of our model and that of Francis for two possibilities of drawn remaining contours, (Figures 4C,D, respectively). In one case, the remaining edges overlap the chromatic gradients (Figure 4, First row), which exist in the inducing stimuli, while in the second case, there is no overlap (Figure 4, second row).

The predictions of both models yielded the same results when the boundaries overlapped (Figure 4, first row, **C,D**), and these results agree with the experimental perceived results reported previously (Kim and Francis, 2011). However, the predictions of the models differed when the boundaries were non-overlapping. Figure 4 second row shows that the inner rectangle is reddish (Figure 4D) according to our model, but gray according to the predictions of Francis' model's (Figure 4C). Notably, the psychophysical findings (Kim and Francis, 2011) support our model, which predicts that remaining contours that do not overlap the chromatic gradient, do not block the diffusion process.

#### Model predictions for a new stimulus with different variations of remaining edges

Having successfully tested our model on previously described stimuli, we proceeded to further challenge the simulations with new spiral stimuli, which have not been described previously or experimentally tested. The new stimuli can simultaneously generate both positive and negative effects because they have both inner and outer borders. This type of stimulus enables us to test a critical property regarding the effect of closed or open remaining edges, on the relevant aftereffects.

**Figure 5:**
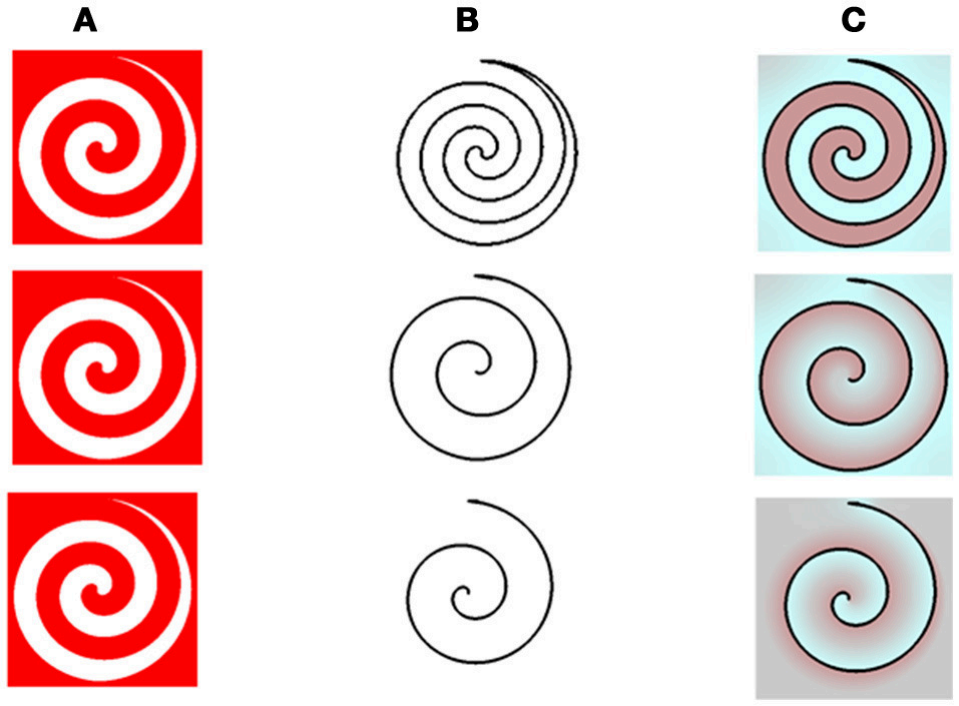
The model predictions for the spiral stimuls with variations in the remaining contours. **(A)** The chromatic stimulus. **(B)** The remaining contours. **(C)** Model's predictions. In this figure, the chromatic stimulus is the same in all the rows, column **(A)**, but the remaining contours are different. In the first row, the drawn contour is a full spiral. In the second row, the outer edge of the spiral shape is presented and in the third row the drawn contour is the inner edge of the spiral shape, column **(B)**. Our model predicts that both cyan and red colors are dominant in the full spiral (first row). When the remaining contour is the outer one, the dominant percieved color is reddish (second row), while, when the remaining contour is on the inner side, the dominant color is cyan (third row).

The model's results for the spiral stimuli, indicated that the dominant color perceived in the afterimage depends on whether the remaining edges are the inner or outer edge, (first and second rows of Figure 5, respectively). The dominant color, predicted by our model, can therefore be either complementary or similar to that of the inducer color, where the outer border produces a dominant positive effect, while the inner border produces a dominant negative effect, (Figure 5C). These predictions are supported by preliminary psychophysical results (Manuscript in preparation).

**Figure 6:**
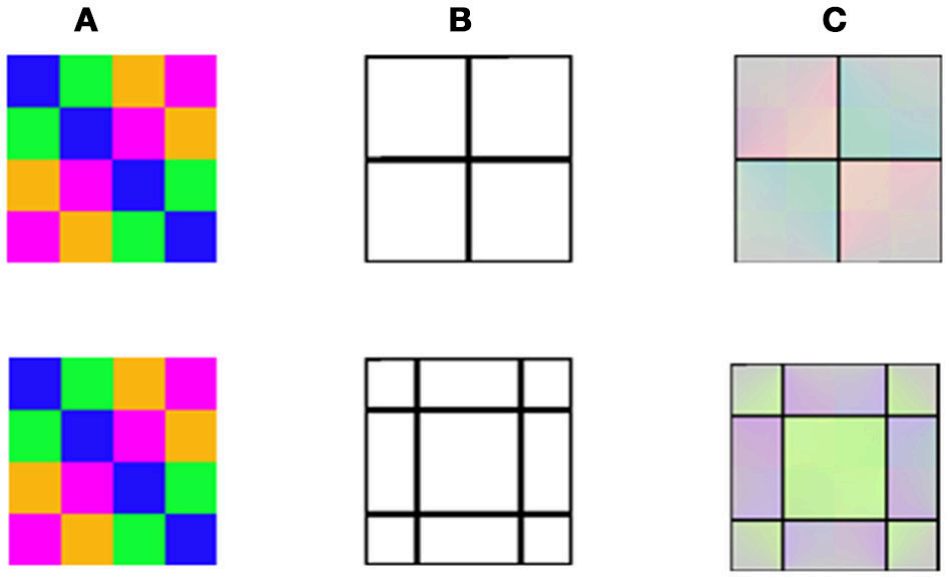
Model predictions for averaging of negative afterimage colors (Anstis et al., 2012). **(A)** The chromatic stimulus. **(B)** The remaining contours with two different locations. **(C)** The model's predictions. It appears that colors of the remaining contours determine the role of color averaging.

**Figure 7:**
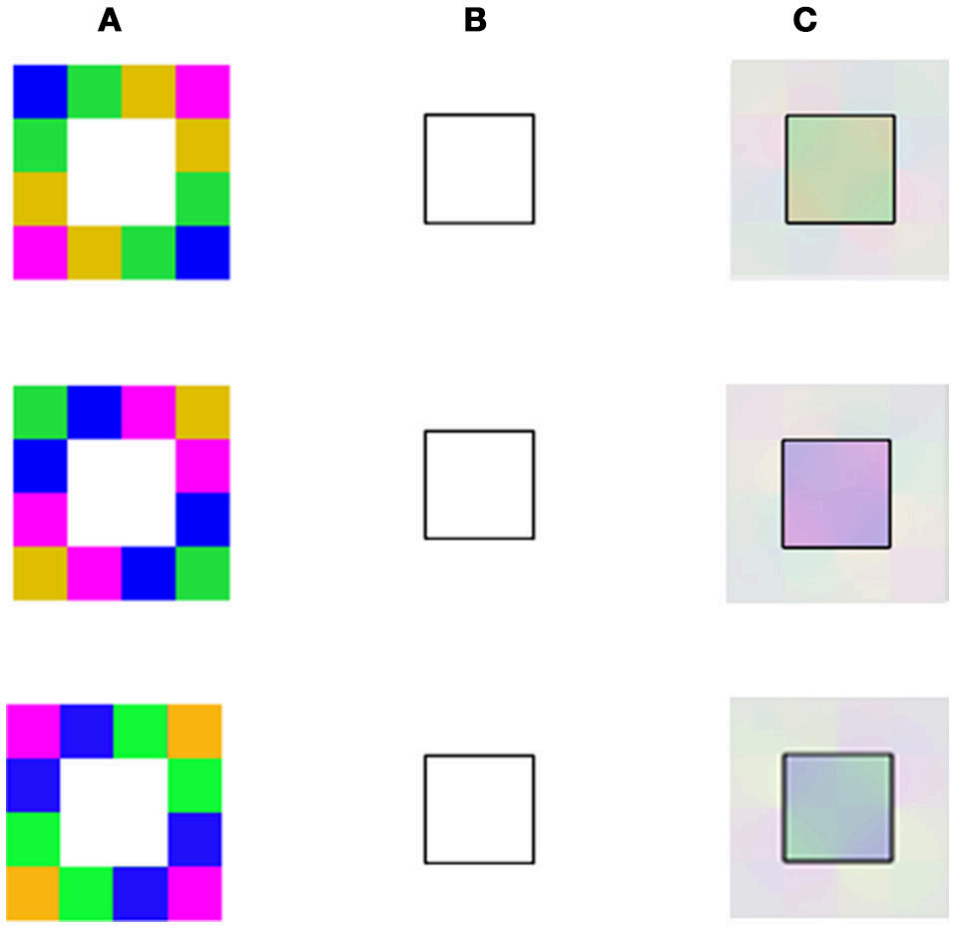
Model predictions for averaging of negative and positive afterimage colors. **(A)** The chromatic stimuli with different color combinations (different rows). **(B)** The remaining contour (identical in all the rows). **(C)** The model's predictions which show that a different “perceived” color is obtained as a result of the chromatic combination of the inducer. It can be seen that there is also an averaging of colors in the positive effect with these new averaging color stimuli.

As a further test, we examined the ability of our model to predict the psychophysical results of the aftereffects that can be perceived from performing spatial averaging within the remaining contours (Anstis et al., 2012). This question was tested by our model simulation under two configurations representing variations of the negative and positive effects (Figures 6, 7). While the negative stimuli are as previously reported (Anstis et al., 2012), the positive stimuli are new and are designed to induce the positive effect. Figures 6, 7 demonstrate the model's predictions for the negative and positive versions of averaging effect, respectively. Note that only the positive configuration (Figure 7) induces a classical filling-in, since this is the only configuration where there is an achromatic area that can be filled with color.

## Discussion

The suggested model involves several stages that can be regarded as a cascade of component mechanisms and responses, i.e., a short duration chromatic stimulus, cessation of this stimulus, creation of complementary chromatic edges which trigger a diffusion process. The suggested model predicts afterimage phenomena, which some of them might appear as “opposite (“conflicting”) effects,” through the same mechanism and therefore the same equations.

We present here a model that is able to predict both the negative and the positive effects, i.e., where the illusionary filled-in color is either the same color or is complementary to that of the inducer. The model, therefore can also predict both the famous “filling-in the afterimage after the image” illusion and the “color dove illusion” (van Lier et al., 2009; Barkan and Spitzer, 2017). In addition, the model can also predict both the positive and the negative versions of the effect in shapes that possess non-closed remaining edges and successfully predicted a recently reported predominantly negative afterimage effect related to averaging of colors (Anstis et al., 2012), Figure 6.

It might be claimed that diffusion models have been previously suggested to predict the aftereffect in general, and also to predict the alternating aftereffect (Grossberg and Mingolla, 1985; Grossberg and Todorovic, 1988; Francis and Rothmayer, 2003; Francis and Ericson, 2004; Francis and Schoonveld, 2005; Wede and Francis, 2006; Van Horn and Francis, 2008). However, in contrast to previous models, such as FACADE, in our model the trigger for the diffusion mechanism is a “heat source,” which implements the diffusion (or heat) equation with a “heat source,” Poisson equation (Weickert, 1998). In other words, in our model, the edges are the only trigger for the diffusion process, and have no other role, for example as direct blockers to the diffusion process, as presented in the FACADE model (Grossberg and Mingolla, 1985; Grossberg and Todorovic, 1988; Francis and Rothmayer, 2003; Francis and Ericson, 2004; Francis and Schoonveld, 2005; Wede and Francis, 2006; Van Horn and Francis, 2008). This difference in rationale between FACADE and our model leads to a different structure of diffusion models, (Equation 7). While the FACADE model is composed of two separated components 1) Boundary contour system (BCS) 2) Feature contour system (FCS), our model is consisted of a single component. This component includes both borders and diffusion mechanism, which are computed in the same process (Equation 7). It is not surprising that such differences give rise to different model predictions in the two types of models, as will be described below.

The model described by Francis (2010) succeeded in predicting the negative effect (van Lier et al., 2009), in which the visual afterimage could spread across regions that were not colored in the inducing stimulus. He also could show, by the application of the FACADE model (Grossberg and Mingolla, 1985), that the perceived color and shape of the afterimage could be manipulated by remaining contours that apparently trapped the spread of afterimage color signals. However, this model also mistakenly predicts that a remaining edge will block the spread of color even if there is no overlap with the chromatic gradient edge border (Figure 1B in: Francis, 2010). This prediction is in disagreement with the psychophysical findings of the experiments conducted by Kim and Francis (2011). In contrast, our simulations indicate that the diffusion process is not blocked when the achromatic remaining contours do not overlap the chromatic contours.

In addition, as already claimed in the introduction, Francis's model cannot predict the positive effect, since his model assumes that the spread of complementary color across a surface will be blocked by the remaining contour. According to the Francis model (Francis, 2010), the positive effect is predicted to be negated, due to the role of the remaining contour which prevents diffusion of the color to the inner part of the shape. Consequently, the model cannot predict the possibility of obtaining result that shows perception of the same color as of the inducer at a different spatial location. Our model, on the other hand, can predict the positive effect (Figure 3), since it assumes that the main role of the contours is to trigger the diffusion process and not primarily aimed to block the diffusion process.

It should be clarified that the FACADE model has been implemented with a number of different diffusion algorithms. Francis, for example, implemented the filling-in process by using a Connected-Component algorithm (Francis and Rothmayer, 2003; Francis, 2010). In the FACADE models the diffusion process is implemented with iterative algorithm, whereas each pixel is averaged with adjacent pixels only if the neighbors are not edges (Grossberg and Todorovic, 1988; Francis and Ericson, 2004; Francis and Schoonveld, 2005; Wede and Francis, 2006; Van Horn and Francis, 2008). In additional studies (Francis and Ericson, 2004; Francis and Schoonveld, 2005) the diffusion model was extended in order to predict additional properties that are related to the MCAI effect. Consequently, the investigators suggested a “non-diffusion” filling-in mechanism, built from directional operations. It has to be noted that in order to predict the MCAI effect a special component was added to the FACADE model, which express the inhibition between orthogonal oriented grids (Francis and Ericson, 2004; Francis and Schoonveld, 2005; Wede and Francis, 2006; Van Horn and Francis, 2008). One important question is whether any of these previous diffusion implementations of the FACADE model (Grossberg and Mingolla, 1985; Grossberg and Todorovic, 1988; Francis and Rothmayer, 2003; Francis and Ericson, 2004; Francis and Schoonveld, 2005; Wede and Francis, 2006; Van Horn and Francis, 2008; Francis, 2010) can successfully predict the positive effect and its variations.

Since the FACADE models mentioned above share the same BCS, which trap the diffusion process and prevent diffusion of the color to the inner part of the shape, they wrongly predict the blockage of the diffusion process in the inner shape, as described experimentally (Kim and Francis, 2011). They also cannot predict the possibility of obtaining the same color as the inducer at different spatial locations and thus cannot predict the positive effect.

While both types of model (ours and the FACADE) assume that the filling-in process is performed by the isomorphic diffusion mechanisms, other groups have suggested that the symbolic mechanism might determine the diffusion process (von der Heydt et al., 2003; Komatsu, 2006; On and van Boxtel, 2017). According to the symbolic theory, “early visual areas extract only the contrast information at the surface border, while the color and shape of the surface are reconstructed in higher areas on the basis of this information” (Komatsu, 2006). Komatsu (2006) reported, however, that neuronal activity of V1 and V2 plays a role in most of the filling-in phenomena such as filling-in at the blind spot, the Craik–O'Brien–Cornsweet illusion, or neon color spreading.

A recent experimental study (On and van Boxtel, 2017) suggested a symbolic mechanism for the negative effect seen in the “stars” of van Lier et al. (2009).” They hypothesized that transparency cues play an important role in the filling-in process of the negative effect and attempted to validate this suggestion through psychophysical experiments. Their results indicated that transparency clues are a prerequisite for the perceived filling-in effect. When the transparency cues were eliminated by removing one color from the star, the new stimulus contained only one color (Figure 1B, in: On and van Boxtel, 2017, Figure 8), and the filling-in effect indeed vanished. However, there is a different and even simpler explanation that can explain their psychophysical results.

**Figure 8:**
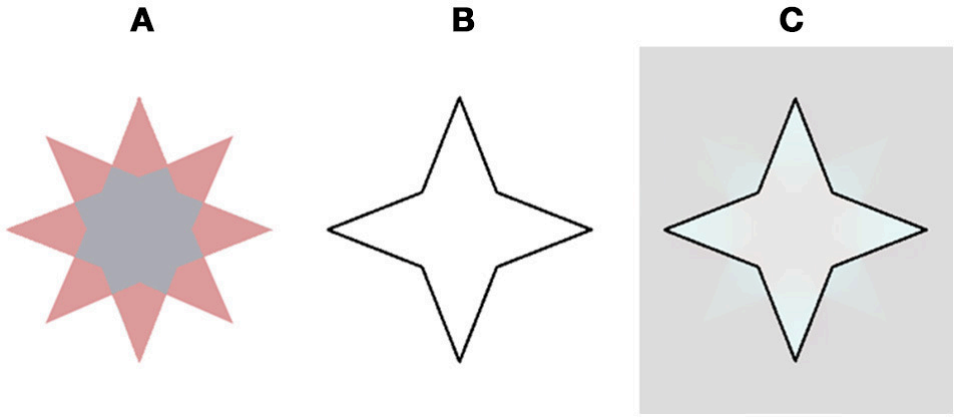
Our model prediction for a star stimulus that does not contains transparency cues and does not yield a filling effect (On and van Boxtel, 2017). This stimulus figured in an experimental study that claimed that the filling does not play a role in the negative aftereffect (Discussion). **(A)** The chromatic stimulus. **(B)** The remaining contour. **(C)** The model's prediction. Note that the complementary color is evident only at the vertices of the star and does not diffuse to the central hexadecagon of the star.

Figure 8 demonstrates our model's prediction for this specific star stimulus. The rationale for this correct prediction is based on the fact that if a combination of the negative and the positive effects act on the same spatial location they cancel each other out, as a result of the simultaneous induction of complementary colors in the same spatial location, Figure 8, (Hazenberg and van Lier, 2013). The original star stimulus of van Lier et al. (2009) consisted of a similar combination of negative and the positive effects, although in this case the two effects enhanced each other. This enhancement was due to the fact that the stars contain two complementary colors (cyan and reddish). When the cyan four-point-star is located inside the remaining contour, the negative effect is produced and the perceived complementary color is reddish. In this case, however, because this reddish four-point-star is located outside the remaining contour, it gives rise to the positive effect, where the perceived color would also be reddish. As a result, the perceived reddish color is enhanced, as a result of the combination of the positive and the negative effects.

It is interesting to consider the stages of analysis of the proposed model as related to components of the visual system. The formation of a complementary or opponent chromatic edge following the cessation of chromatic stimulus (Figure 2) has recently been described in the literature as being attributable to a rebound response (Off response), evoked as a burst of spikes from neurons released from the period of inhibition (Spitzer et al., 1993; Grunfeld and Spitzer, 1995; Francis, 2010; Zaidi et al., 2012). The mechanism by which this produces the perception of the complementary color was suggested to be through cross inhibition between opponent channels (Grossberg, 1972; Francis, 2010), or through fast adaptation from the first order (Spitzer and Semo, 2002; Spitzer and Barkan, 2005). The mechanism suggested for the rebound model of Grunfeld and Spitzer (1995) includes the parameters required for the rebound effect, such as the duration of adaptation, the rate and the intensity of the offset of the stimulus. The current model does not include these additional stimulus parameters, but we plan to include these parameters in future.

The development of a further stage of the model has to be discussed in relation to the visual system and to other models. After the rebound response creates the complementary color, the diffusion process is triggered by different components in each model. According to the FACADE model (Grossberg and Mingolla, 1985; Grossberg and Todorovic, 1988; Francis and Rothmayer, 2003; Francis and Ericson, 2004; Francis and Schoonveld, 2005; Wede and Francis, 2006; Van Horn and Francis, 2008), the trigger for the diffusion process is the color of the surface at each location. This was described as “color spreads all across the surface within the boundary” (Kim and Francis, 2011). In contrast, in our model, the borders (the chromatic edges, i.e., double opponent, in the chromatic stimulus and the remaining contours, as a modulation to the chromatic edges) are the trigger for the diffusion process (Equation 7).

The experimental results of Hazenberg and van Lier (2013) appear to support our model with regard to the trigger for the diffusion process. These researchers demonstrated experimentally that the location of remaining contour that overlaps the chromatic edge can determine whether the result will be a positive or a negative effect. In fact, our model suggests that the perceived chromatic edge triggers an isomorphic filling-in process, according to isomorphic filling-in theory (von der Heydt et al., 2003). It should be noted that the idea that an afterimage of the chromatic contours triggers the isomorphic diffusion process has been raised previously by Hazenberg and van Lier (2013). It has also been suggested that the color signals in this type of filling-in process, spread in all directions except across borders formed by contour activity (Gerrits and Vendrik, 1970; Cohen and Grossberg, 1984; Arrington, 1994; von der Heydt et al., 2003). The role of the remaining contour is therefore in agreement with the previous suggestion that the contours act as diffusion barriers (Cohen and Grossberg, 1984; von der Heydt et al., 2003). However, according to the current model, this remaining contour is effective as a barrier only when it overlaps with the original chromatic edge of the inducer stimulus. Our model therefore suggests that the remaining contour fulfills two functions: a. enhancing the effect of the inverted chromatic edge Equation (4), b. trapping the diffusion. This dual role is supported by the isomorphic filling-in theory of von der Heydt et al. (2003) who suggested that the chromatic or achromatic receptive field plays a role in the filling-in process. The chromatic-edge receptive fields receive additional activation through horizontal connections (Gilbert and Wiesel, 1979), which keep the border activity high. Their suggestion is general and was not specifically related to the visual effects discussed here (the positive and negative effects).

In addition to the crucial role of the remaining contour, which overlap the chromatic gradients, the chromatic edges (by themselves) also play a role in the perceived afterimage, (Equation 11). This assumption was supported by the findings of Hazenberg and van Lier (2013), who reported that the filling-in process, (in their version for the positive effect), should be influenced less by the chromatic gradients (Anstis et al., 1978; Hazenberg and van Lier, 2013).

Since the model takes into account the role of the chromatic edges, albeit with less weight than the remaining contour, it predicts that the diffusion at the negative effect will be partially blocked by the original chromatic gradient of the inducing stimulus. As a result, it predicts that the diffusion will not spread to the central area in the negative effect stimuli, Figure 3.

Our model predicts that if a border does not exist in the original inducing stimulus, it will not block the diffusion process, as found psychophysically (Kim and Francis, 2011). After conducting psychophysical experiments, Kim and Francis (2011) formulated a qualitative rule that additional contours block color spreading when these contours overlap the inducer edges, but not when they are separated (Supplementary Figure 1). It has to be noted that our model's predictions of these results also agree with the qualitative arguments of Hazenberg and van Lier (2013) that there has to be a match (or overlap) between the chromatic edges and the remaining contours. This is derived from a repeated activation of orientation selective neurons that also code for color (von der Heydt et al., 2003).

We also investigated the question of whether it is necessary for the remaining contour to be closed or whether an open spiral stimulus, (Figure 5) can produce the effect. Preliminary results are in agreement with our model predictions that the effect can exist in open boundary conditions (Figure 5). It should be noted that Francis's simulations cannot predict the negative effect on open boundary conditions, such as in the spiral stimulus (Figure 5), because his model depends on a boundary that traps the spread of color (Francis, 2010). However, by applying a previous diffusion model as in Grossberg and Todorovic (1988), a correct prediction can be achieved, but only for the negative parts of the spiral illusion (i.e., only the configuration where the inner border of the spiral is displayed, third row of Figure 5). This is because this case involves a difusion process rather than a Connected Component algorithm as in the Francis implementation (Francis and Rothmayer, 2003; Francis, 2010). However, this modification still cannot predict the positive effect in the spiral illusion (second row of Figure 5).

A further question was whether the aftereffects can be perceived from spatial averaging within the area of remaining contours. Anstis et al. (2012) showed that colors can undergo spatial averaging within, but not across, contours but tested this effect only on the negative aftereffect. Our model's simulations (Figures 6, 7) are with agreement to the experiments conducted by Anstis et al. (2012). We believe that even if the Francis model was able to predict this averaging effect, it could only work on the negative configuration of the effect.

Our results thus far suggest that the same basic mechanism is responsible for both the negative and the positive effects, but there remains a question as to whether there are additional mechanism's components that differentiate between these two mechanisms. The recent study of Hazenberg and van Lier (2013) can shed a light on this issue, since they investigated several properties of the positive and the negative effects on the afterimage watercolor stimuli. Specifically, they examined the role of the intensity of the inner area of the inducer stimulus and the remaining contour with reference to the positive and the negative effects.

The results of their study indicated that the filling-in effect was stronger in the negative effect under conditions where the inner area of the inducer stimulus was gray (iso-luminance with the chromatic borders) rather than white. This preference was not found in the positive effect. Hazenberg and van Lier (2013) interpreted these findings as the result of the influence of the luminance border between the inner chromatic contour and the interior area. This luminance border was presumed to prevent the colored afterimage of the chromatic contour from spreading. However, under iso-luminance conditions, the luminance borders do not exist, and indeed, the filling-in process is more prominently perceived. Our model can be modified, by taking into account a combination of the chromatic and the achromatic gradients of the chromatic stimulus, in order to predict this influence on the inner area intensity. Due to the differences related to the positive and negative effects, our model predicts that the negative effect will be more prominent with regard to the degree of saturation, while the positive effect will be more prominent in its ability to perform a filling-in task. This prediction should be confirmed by psychophysical experiments.

In order to test the role of the intensity of the remaining contour Hazenberg and van Lier (2013) used thick contours colored either light or dark gray as the remaining contours. They reported that the filling-in effect was perceived only when the contours were gray and not black, and only in case of the positive effect (i.e., where the perceived color is the same as the inducer).

We now suggest that according to our model (Figure 3), both gray and black contours can create a complementary color effect, but only in the near vicinity of the chromatic border in the original chromatic stimulus. It is possible that the lack of filling-in color in the positive effect (Figure 8 in: Hazenberg and van Lier, 2013), was a consequence of the contour thickness of the remaining contours, since in the positive effect, the color has to diffuse through the remaining contour. The border contrast with a gray contour is weaker, and therefore reveals a partial filling-in effect. We suggest that the negative effect was not observed in the reported experiments (Hazenberg and van Lier, 2013) because they were looking mainly at the central area of the stimulus. Such a filling-in color is not expected in the inner white area (Figure 7 in: Hazenberg and van Lier, 2013) because it is blocked by the luminance border, which contributes to the blockage of the filling-in process [Equation (7), Figure 3].

Additional factors that might affect the degree of the aftereffect e.g., include the size of the inducer and induced area, the shape curvature of the chromatic edge, and the exposure duration of the chromatic stimulus. These factors should be separately investigated experimentally for their influence on the positive and the negative effects. Psychophysical experiments are important in order to detect differences in the mechanisms acting in these two types of effects. In addition, psychophysical experiment are required for cases where the remaining contours that trigger the filling-in effect are illusory contours, such as those in the Kanizsa effect and the Neon color spreading effects (Van Tuijl, 1975; Kanizsa, 1976). This should be tested separately for the positive and negative effects. Our model predicts that for an illusory contour stimulus (which replaces the achromatic remaining contour), the chromatic and the illusory remaining contour have to overlap. However, we believe that the mechanism which creates the illusory contour (as produced in a Kanizsa illusion) is different from the filling-in mechanism. [Different computational models suggested in the literature for the Kanizsa illusion (Grossberg and Mingolla, 1985, 1987; Heitger et al., 1998; Ron and Spitzer, 2011)]. In order to include the prediction of the filling-in effect triggered by illusory contours, we will need to combine the different mechanisms of the illusory contours and the filling-in mechanism, and will therefore need to add an additional model component to detect the illusory contours.

The MCAI Effect (MacKay, 1957; Vidyasagar et al., 1999) is an alternating aftereffect but it differs from the positive and the negative aftereffects, as it contains an additional component, which relates to a different mechanism. This component enables oriented adaptation in the MCAI oriented stimulus (more specifically, of the flickering grid in the relevant stimulus). We expect that our filling-in model will predict this MCAI effect, but only if an additional component, which describes such oriented adaption mechanism (of the MCAI effect), will be added to the model.

Even though the present model does not permit predictions of the behavior of all the free parameters that play a role in the negative and positive effects, this is the first time that a computational model has been able to make crucial predictions on both the positive and the negative effects. In other words, our model succeeds in predicting apparently conflicting phenomena, i.e., those producing the complementary or same color aftereffect, and implies that the same mechanisms function in both effects despite the different manifestations. An important conclusion of this study is that a different appearance does not necessarily infer a difference in the causative mechanisms and driving forces.

The proposed model has several possible applications with the potential to be an applicable algorithm for the restoration of corrupted old images and videos, for example. Such an algorithm may be able to make an educated guess for filling-in color, based on partial information, such as having only remaining contours.

## Author contributions

All authors listed have made a substantial, direct and intellectual contribution to the work, and approved it for publication.

### Conflict of interest statement

The authors declare that the research was conducted in the absence of any commercial or financial relationships that could be construed as a potential conflict of interest.

## References

[B1] Anstis S. Rogers B. Henry J. (1978). Interactions between simultaneous contrast and coloured afterimages. Vis. Res. 18, 899–911. 10.1016/0042-6989(78)90016-0 706164

[B2] Anstis S. Vergeer M. Van Lier R. (2012). Luminance contours can gate afterimage colors and “real” colors. J. Vis. 12:2. 10.1167/12.10.2 22961219

[B3] Arrington K. F. (1994). The temporal dynamics of brightness filling-in. Vis. Res. 34, 3371–3387. 10.1016/0042-6989(94)90071-X 7863620

[B4] Barkan Y. Spitzer H. (2009). Color Dove Illusion | Best Illusion of the Year Contest. Available online at: http://illusionoftheyear.com/2009/05/color-dove-illusion/

[B5] Barkan Y. Spitzer H. (2017). The color dove illusion- chromatic filling in effect following a spatial-temporal edge, in The Oxford Compendium of Visual Illusions, eds Shapiro A. G. Todorovic D. (Oxford; New York, NY: Oxford University Press), 752–755.

[B6] Barkan Y. Spitzer H. Einav S. (2008). Brightness contrast-contrast induction model predicts assimilation and inverted assimilation effects. J. Vis. 8, 27–27. 10.1167/8.7.27 19146233

[B7] Clair R. S. Hong S. W. Shevell S. (2007). Misbinding of color to form in afterimages. J. Vis. 7, 366–366. 10.1167/7.9.366 18321397

[B8] Cohen M. A. Grossberg S. (1984). Neural dynamics of brightness perception: features, boundaries, diffusion, and resonance. Percept. Psychophys. 36, 428–456. 10.3758/BF03207497 6398424

[B9] Cohen-Duwek H. Spitzer H. (2017). A new diffusion computational model predicts both the positive and the negative short afterimage effects, in Color and Imaging Conference (Springfield, VA: Society for Imaging Science and Technology), 103–107.

[B10] Conway B. R. (2001). Spatial structure of cone inputs to color cells in alert macaque primary visual cortex (V-1). J. Neurosci. 21, 2768–2783. 10.1523/JNEUROSCI.21-08-02768.2001 11306629 PMC6762533

[B11] Conway B. R. Livingstone M. S. (2006). Spatial and temporal properties of cone signals in alert macaque primary visual cortex. J. Neurosci. 26, 10826–10846. 10.1523/JNEUROSCI.2091-06.2006 17050721 PMC2963176

[B12] Daw N. W. (1962). Why after-images are not seen in normal circumstances. Nature 196, 1143–1145. 10.1038/1961143a0 14025557

[B13] DeValois K. Webster M. (2011). Color vision. Scholarpedia 6:3073 10.4249/scholarpedia.3073

[B14] Francis G. (2010). Modeling filling-in of afterimages. Atten. Percept. Psychophys. 72, 19–22. 10.3758/APP.72.1.19 20045876

[B15] Francis G. Ericson J. (2004). Using afterimages to test neural mechanisms for perceptual filling-in. Neural Netw. 17, 737–752. 10.1016/j.neunet.2004.01.007 15288895

[B16] Francis G. Rothmayer M. (2003). Interactions of afterimages for orientation and color: experimental data and model simulations. Percept. Psychophys. 65, 508–522. 10.3758/BF03194579 12812275

[B17] Francis G. Schoonveld W. (2005). Using afterimages for orientation and color to explore mechanisms of visual filling-in. Percept. Psychophys. 67, 383–397. 10.3758/BF03193319 16119389

[B18] Gerrits H. J. M. Vendrik A. J. H. (1970). Simultaneous contrast, filling-in process and information processing in man's visual system. *Exp*. Brain Res. 11, 411–430. 10.1007/BF00237914 5496938

[B19] Gilbert C. D. Wiesel T. N. (1979). Morphology and intracortical projections of functionally characterised neurones in the cat visual cortex. Nature 280, 120–125. 10.1038/280120a0 552600

[B20] Grossberg S. (1972). A neural theory of punishment and avoidance, II: quantitative theory. Math. Biosci. 15, 253–285. 10.1016/0025-5564(72)90038-7

[B21] Grossberg S. Mingolla E. (1985). Neural dynamics of form perception: boundary completion, illusory figures, and neon color spreading. Psychol. Rev. 92, 173–211. 10.1037/0033-295X.92.2.173 3887450

[B22] Grossberg S. Mingolla E. (1987). Neural dynamics of perceptual grouping: textures, boundaries, and emergent segmentations, in The Adaptive Brain, II, (Elsevier), 143–210. 10.3758/bf03198851 4088806

[B23] Grossberg S. Todorovic D. (1988). Neural dynamics of 1-D and 2-D brightness perception: a unified model of classical and recent phenomena. Percept. Psychophys. 43, 241–277. 10.3758/BF03207869 3347487

[B24] Grunfeld E. D. Spitzer H. (1995). Spatio-temporal model for subjective colours based on colour coded ganglion cells. Vis. Res. 35, 275–283. 10.1016/0042-6989(94)00119-7 7839622

[B25] Hazenberg S. J. van Lier R. (2013). Afterimage watercolors: an exploration of contour-based afterimage filling-in. Front. Psychol. 4:707. 10.3389/fpsyg.2013.00707 24115940 PMC3792352

[B26] Heitger F. von der Heydt R. Peterhans E. Rosenthaler L. Kübler O. (1998). Simulation of neural contour mechanisms: representing anomalous contours. Image Vis. Comput. 16, 407–421. 10.1016/S0262-8856(97)00083-8 1604865

[B27] Kandel E. R. Schwartz J. H. Jessell T. M. Siegelbaum S. A. Hudspeth A. J. (2012). Principles of Neural Science, 5th Edn. New York, NY: McGraw-Hill Education/Medical.

[B28] Kanizsa G. (1976). Subjective contours. Sci. Am. 234, 48–52. 10.1038/scientificamerican0476-48 1257734

[B29] Kim J. Francis G. (2011). Color selection, color capture, and afterimage filling-in. J. Vis. 11, 23–23. 10.1167/11.3.23 21454856

[B30] Komatsu H. (2006). The neural mechanisms of perceptual filling-in. Nat. Rev. Neurosci. 7, 220–231. 10.1038/nrn1869 16495943

[B31] MacKay D. M. (1957). Moving visual images produced by regular stationary patterns. Nature 180, 849–850. 10.1038/180849a0 13504203

[B32] Macknik S. L. Martinez-Conde S. (2010). The Neuroscience of Yoricks's Ghost and Other Afterimages. Sci. Am. 20, 12–15.

[B33] Marr D. (1982). Vision: A Computational Approach. New York, NY: Freeman.[aAC].

[B34] On Z. X. van Boxtel J. J. (2017). The role of transparency cues in afterimage color perception. Sci. Rep. 7:9183. 10.1038/s41598-017-09186-1 28835656 PMC5569075

[B35] Pérez P. Gangnet M. Blake A. (2003). Poisson image editing, in ACM SIGGRAPH 2003 Papers (New York, NY: ACM), 313–318.

[B36] Ron E. Spitzer H. (2011). Is the Kanizsa illusion triggered by the simultaneous contrast mechanism? J. Opt. Soc. Am. A Opt. Image Sci. Vis. 28, 2629–2641. 10.1364/JOSAA.28.002629 22193276

[B37] Sande K. van de Gevers T. Snoek C. (2010). Evaluating color descriptors for object and scene recognition. IEEE Trans. Pattern Anal. Mach. Intell. 32, 1582–1596. 10.1109/TPAMI.2009.154 20634554

[B38] Shapley R. Hawken M. (2011). Color in the cortex—single- and double-opponent cells. Vis. Res. 51, 701–717. 10.1016/j.visres.2011.02.012 21333672 PMC3121536

[B39] Shevell S. K. (2003). The Science of Color. New York, NY: Elsevier.

[B40] Shimojo S. Kamitani Y. Nishida S. (2001). Afterimage of perceptually filled-in surface. Science 293, 1677–1680. 10.1126/science.1060161 11533495

[B41] Simchony T. Chellappa R. Shao M. (1990). Direct analytical methods for solving poisson equations in computer vision problems. IEEE Trans. Pattern Anal. Mach. Intell. 12, 435–446. 10.1109/34.55103

[B42] Spitzer H. Almon M. Sandler V. M. (1993). A model for detection of spatial and temporal edges by a single X cell. Vis. Res. 33, 1871–1880. 10.1016/0042-6989(93)90178-Y 8266643

[B43] Spitzer H. Barkan Y. (2005). Computational adaptation model and its predictions for color induction of first and second orders. Vis. Res. 45, 3323–3342. 10.1016/j.visres.2005.08.002 16169037

[B44] Spitzer H. Semo S. (2002). Color constancy: a biological model and its application for still and video images. Pattern Recognit. 35, 1645–1659. 10.1016/S0031-3203(01)00160-1

[B45] Van Horn D. R. Francis G. (2008). Orientation tuning of a two-stimulus afterimage: implications for theories of filling-in. Adv. Cogn. Psychol. 3, 375–387. 10.2478/v10053-008-0002-7 20517521 PMC2864993

[B46] van Lier R. Vergeer M. Anstis S. (2009). Filling-in afterimage colors between the lines. *Curr*. Biol. 19, R323–R324. 10.1016/j.cub.2009.03.010 19409278

[B47] Van Tuijl H. (1975). A new visual illusion: neonlike color spreading and complementary color induction between subjective contours. Acta Psychol. 39, 441–445. 10.1016/0001-6918(75)90042-6 1199780

[B48] Vidyasagar T. R. Buzás P. Kisvárday Z. F. Eysel U. T. (1999). Release from inhibition reveals the visual past. Nature 399:422. 10.1038/20836 10365954

[B49] von der Heydt R. Friedman H. S. Hong Z. (2003). Searching for the neural mechanisms of color filling-in, in Filling-In : From Perceptual Completion to Cortical Reorganization: From Perceptual Completion to Cortical Reorganization, eds Pessoa L. Weerd P. (Oxford, UK: Oxford University Press), 106–127.

[B50] Webster M. A. (2015). Visual adaptation. Annu. Rev. Vis. Sci. 1, 547–567. 10.1146/annurev-vision-082114-035509 26858985 PMC4742349

[B51] Wede J. Francis G. (2007). Attentional effects on afterimages: theory and data. Vision Res. 47, 2249–2258. 10.1016/j.visres.2007.04.024 17610930

[B52] Wede J. Francis G. (2006). The time course of visual afterimages: data and theory. Perception 35, 1155–1170. 10.1068/p5521 17120838

[B53] Weickert J. (1998). Anisotropic Diffusion in Image Processing. Stuttgart: Teubner.

[B54] Williams D. R. Macleod D. I. (1979). Interchangeable backgrounds for cone afterimages. Vis. Res. 19, 867–877. 10.1016/0042-6989(79)90020-8 316228

[B55] Wyszecki G. (1986). Color appearance, in Handbook of Perception and Human Performance, eds Boff K. R. Kaufman L. Thomas J. P. (New York, NY: John Wiley & Sons), 29–30.

[B56] Zaidi Q. Ennis R. Cao D. Lee B. (2012). Neural locus of color afterimages. Curr. Biol. 22, 220–224. 10.1016/j.cub.2011.12.021 22264612 PMC3562597

[B57] Zeki S. Cheadle S. Pepper J. Mylonas D. (2017). The constancy of colored after-images. Front. Hum. Neurosci. 11:229. 10.3389/fnhum.2017.00229 28539878 PMC5423953

