# Supplementary material for: A Model for a Filling-in Process Triggered by Edges Predicts “Conflicting” Afterimage Effects
Source: Front Neurosci. 2018 Aug 17;12:559. doi: 10.3389/fnins.2018.00559 (PMC6107801; doi:10.3389/fnins.2018.00559)
Supplement: Supplementary file 1 [file Data_Sheet_1.PDF]

Supplementary Material

A model for a filling-in process triggered by edges predicts "conflicting" afterimage effects

Hadar Cohen-Duwek[1], Hedva Spitzer[1]

*** Correspondence:** Corresponding Author: hadarli@gmail.com

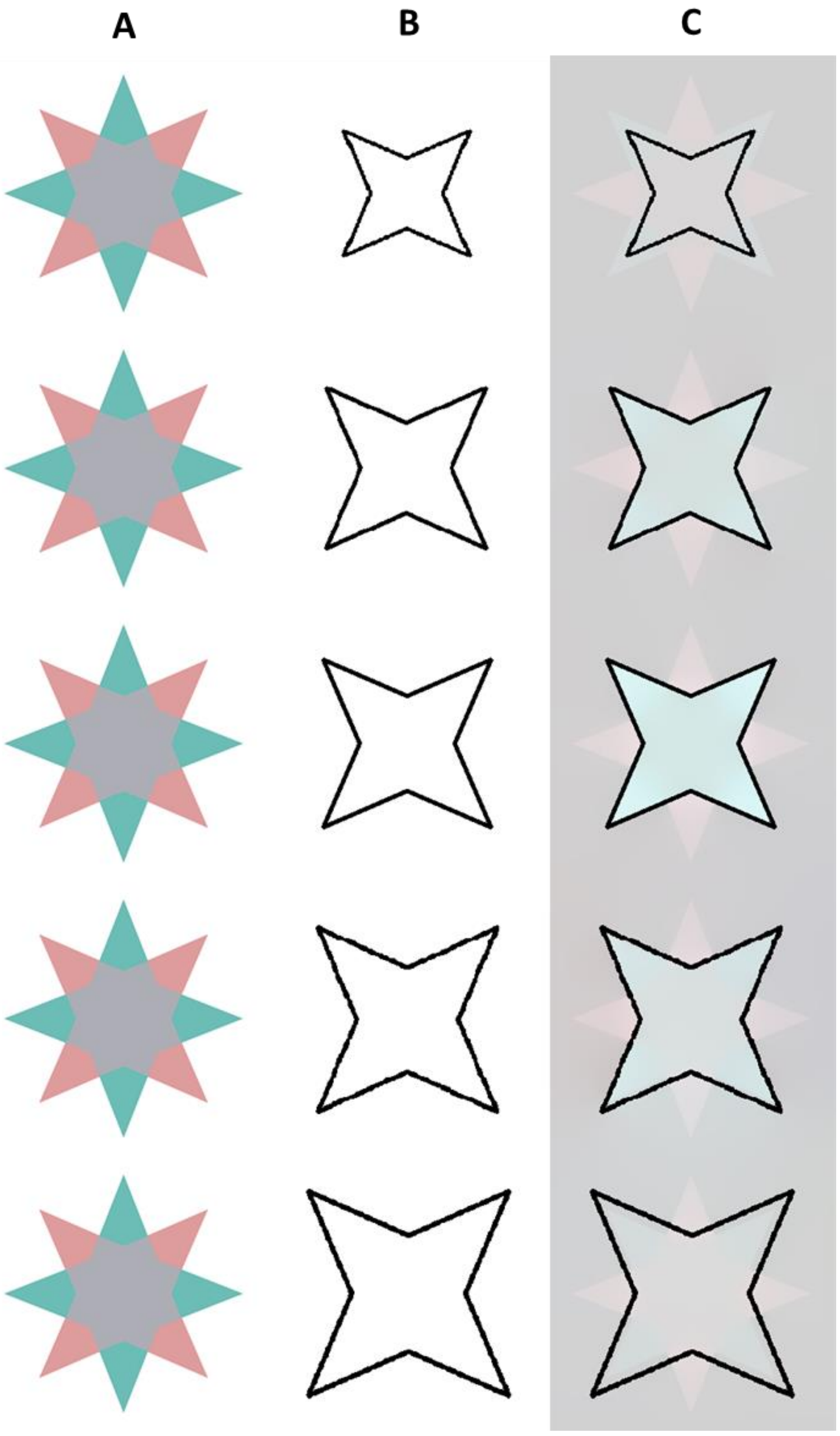

Supplementary Figure 1. The suggested model's predictions to experimant 3 of Kim and Francis, (2011). A) The chromatic stimulus. B) The remaining contours. C) The simulation results. The results demonstrate that: "These results imply that close proximity between the inducer and the contour enhanced the afterimage perception". (Kim & Francis 2011). Parameters:α=1.3 and β=0.3. Note that the value of β, in the above results, is slightly ehanhanced for this demonstration
